# Prognostic and predictive factors in pancreatic cancer

**DOI:** 10.18632/oncotarget.27518

**Published:** 2020-03-10

**Authors:** Emanuela Dell’Aquila, Claudia Angela Maria Fulgenzi, Alessandro Minelli, Fabrizio Citarella, Marco Stellato, Francesco Pantano, Marco Russano, Maria Concetta Cursano, Andrea Napolitano, Tea Zeppola, Bruno Vincenzi, Giuseppe Tonini, Daniele Santini

**Affiliations:** ^1^ Department of Medical Oncology, University Campus Bio-Medico, Rome 00128, Italy

**Keywords:** metastatic pancreatic cancer, predictive and prognostic factors, CA19-9, gemcitabine-abraxane, FOLFIRINOX

## Abstract

Pancreatic cancer is one of the leading causes of cancer death worldwide. Its high mortality rate has remained unchanged for years. Radiotherapy and surgery are considered standard treatments in early and locally advanced stages. Chemotherapy is the only option for metastatic patients. Two treatment regimens, i. e. the association of 5-fluorouracil- irinotecan-oxaliplatin (FOLFIRINOX) and the association of nab-paclitaxel with gemcitabine, have been shown to improve outcomes for metastatic pancreatic adenocarcinoma patients. However, there are not standardized predictive biomarkers able to identify patients who benefit most from treatments. CA19-9 is the most studied prognostic biomarker, its predictive role remains unclear. Other clinical, histological and molecular biomarkers are emerging in prognostic and predictive settings. The aim of this review is to provide an overview of prognostic and predictive markers used in clinical practice and to explore the most promising fields of research in terms of treatment selection and tailored therapy in pancreatic cancer.

## INTRODUCTION

Pancreatic ductal adenocarcinoma (PDAC) is the 12th most frequent cancer in the world and it is the 4th cause of cancer-related death in Western Countries, with a mortality rate almost equal to its incidence [1] and a 5 year survival rate of 5–7% [2]. The vast majority of patients is diagnosed with metastatic or inoperable disease due to the vagueness of symptoms in the initial stages.

Radiotherapy and surgery can be considered in early stage or locally advanced disease [3, 4], while chemotherapy is the current standard of care in metastatic setting [5, 6].

FOLFIRINOX or gemcitabine plus nab-paclitaxel are used in first-line for metastatic PDAC (mPDAC) [5]. In clinical practice there are not clear predictive and prognostic factors that aid in choosing the best regimen for every patient balancing wisely between the benefit and the drug related toxicities. Generally, fit patients are treated with FOLFIRINOX, whereas older and unfit patients receive gemcitabine plus nab-paclitaxel or mono-chemotherapy.

In this review, we provide an overview of available evidences on prognostic and predictive clinical and biological markers in mPDAC that could help clinicians in treatment decision.

Over the last decades, a number of prognostic and predictive factors has been evaluated in mPDAC.

**Figure 1:**
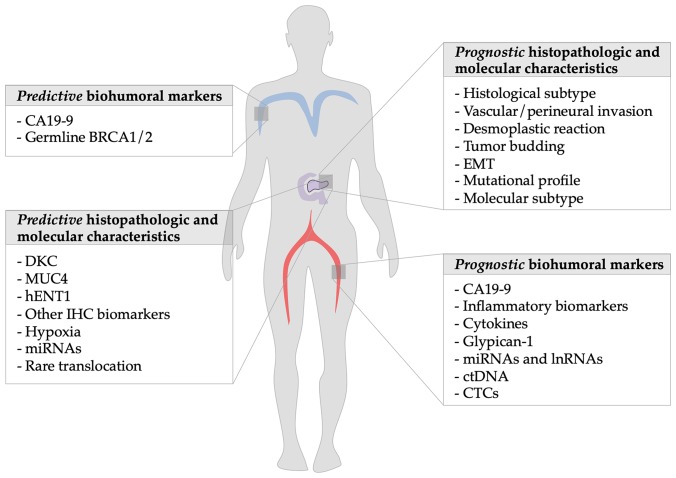
Summary of prognostic factors of metastatic pancreatic ductal adenocarcinoma: The figure shows on the left side the predictive markers discussed in the article and on the right the prognostic ones.

Among these, we will analyze the evidences on histopathological characteristics, clinical and biohumoral markers (see Tables 1 and 2), with a particular focus on CA19-9, markers of systemic inflammation and immune-modulation, and novel molecular surrogates of survival (Figure 1).

**Table 1:** Promising prognostic and predictive biomarkers

Authors	Markers investigated	Role	Prognostic role
Lu *et al*. [40]	Glypican-1 (GPC1)-expressing circulating exosomes	Involvement in angiogenesis and tumor growth	GPC1 overexpression associated with poorer OS
Giovannetti *et al*. [46]	Circulating miRNA 21	Modulation of apoptosis, Akt phosphorylation, and expression of genes involved in invasive behavior	High miR-21 expression predicted shorter OS both in the metastatic and in the adjuvant setting
Korpal *et al*. [50]	miRNA 200 family	Loss of expression of miRNA-200 family members may play a critical role in enhancing migration and invasion during cancer progression	Not investigated in clinical studies
Liu *et al*. [56]	lncRNA MALAT1	Identified in multiple types of physiological and pathological processes, i. e. organizing nuclearconstruction and modulating gene expression	Higher expression of lncRNA MALAT1 was associated with poorer OS in patients affected by PDAC
Ye *et al*. [57]	lncRNA AFAP1-AS1	Knockdown of AFAP1-AS1 could inhibit cell proliferation, migration, and invasion of PDAC cells	LncRNA AFAP1-AS1 overexpression was associated with lymph node metastasis, perineural invasion, and poor survival
Creemers *et al*. [70]	CtDNA	N/A	ctDNA is associated with poor prognosis in patients with pancreatic cancer

The table summarizes the most relevant studies that investigated the prognostic or predictive significance of novel biomarkers.

**Table 2:** Correlation between inflammatory markers and prognosis

Authors	Markers investigated	Study design and setting	Results
Martin *et al*. [90]	NLR; PLR; mGPS	Retrospective analysis of 124 patients with PDAC (84 mPDAC)	NLR; PLR and mGPS resulted independent prognostic markers
Stotz *et al*. [96]	NLR; mGPS, PLR	Retrospective evaluation of 271 inoperable patients with PDAC	NLR>5 and mGPS of 1-2 predicted poorer OS
Liu *et al*. [97]	CRP/Albumin ratio	Retrospective analysis of 386 patients with PDAC (174 mPDAC)	CRP/Albumin ratio >0.180 predicted poorer OS
Yamada *et al*. [98]	Neutropenia	Retrospective analysis of patients treated with mFOLFIRINOX for mPDAC	Patients with neutropenia after chemotherapy experienced better survival
Yu *et al*. [100]	LDH; LMR; NLR; PLR; Ca 19-9	Patients with inoperable PDAC treated with Gemcitabine based chemotherapy	Ca 19-9 ≥1000 IU/mL; LDH ≥185; NLR≥3.42; LMR≥3.19 predicted poorer OS
Hwang *et al*. [103]	mGPS; NLR; PLR	Patients treated with Gemcitabine and Nab-Paclitaxel for mPDAC	mGPS≥1 predicted poorer OS; NLR and PLR did not predict poorer OS

NLR: neutrophil to lymphocyte ratio; mGPS: modified Glasgow Prognostic Score; PLR: Platelet to lymphocyte ratio; LMR: *lymphocyte* to *monocyte* ratio.

## HISTOPATHOLOGICAL CHARACTERISTICS

Histological analysis of primary tumor or metastatic sites is the only approved diagnostic tool for pancreatic cancer. Aside from histological diagnosis, tissue analysis has been largely used to identify prognostic features that could influence treatment decision.

In patients undergoing surgery, the histological analysis defines the pathological stage according to the 8^th^ edition of the TNM system [7]. The TNM staging system relies on its reproducibility using anatomic parameters to stratify patients with different survival outcomes [8]. Differently from the 7th edition, the current TNM system does not consider the extension outside the pancreas as T3, because staging in the T stage has been replaced by a size-based system [8]. The N (nodes) parameter has been divided in N1 and N2 depending on the number of positive regional lymph nodes. The number of lymph nodes involved remains the strongest predictor of survival in operable patients [9].

Based on morphological features, different PDAC subtypes have been identified.

Variants with a similar molecular pathogenesis include, among others, adenosquamous carcinoma, anaplastic (undifferentiated) carcinoma and undifferentiated carcinoma with osteoclastic giant cells. Adenosquamous and anaplastic carcinomas carry worse prognosis than classical PDAC [10]. On the other hand, colloid, medullary carcinoma and hepatoid adenocarcinoma are variants with a distinct molecular pathogenesis. Considering their low incidence, the prognostic significance of these pathological characteristic is yet to be defined.

Other prognostic factors identified through pathological analysis of surgical samples are vascular and perineural invasion [11], presence of desmoplastic reaction [12], tumor budding and epithelial to mesenchymal transition (EMT) [13, 14].

PDAC produces a strong fibrotic reaction around the primary tumor [12], known as desmoplasia, typical of many malignancies. The fibrotic tissue plays an important role in tumorigenesis, angiogenesis, and resistance to therapy [12]. Cancer-associated fibroblasts (CAFs) are the main effector cells in the desmoplastic reaction, and pancreatic stellate cells (PSCs) are the most important source of CAFs [15]. CAFs have been reported to promote PDAC cell growth, to stimulate stroma production by PSCs and their presence in peritumoral stroma has been associated with worse prognosis [16]. Furthermore, activated CAFs have been implicated in chemo-radiation resistance, for those reasons, therapeutic strategies to target stromal cells are under investigation [15]. CAFs can determine chemotherapy resistance through several molecular mechanisms including upregulation of genes involved in extracellular matrix synthesis (SPARC) and in the transduction of chemokines signal (CXCL12/CXCR4), resulting in the induction of epithelial–mesenchymal transition (EMT) [17].

Tumor budding is defined as the presence of detached isolated single cells or small cell clusters (up to five cells) scattered in the stroma at the invasive tumor margin [18]. The presence of tumor budding in surgical samples has been clearly described to be related to advanced pT classification, lymphatic invasion, decreased disease-free and overall survival (DFS; OS) [13]. A correlation between the grade of tumor budding and the immune system invasion has been reported, with high grade of tumor budding being associated with FOXP3+ regulatory T cells invasion and worse prognosis [19].

Interestingly, an interplay between the presence of tumor budding and the development of EMT is emerging. Experimental data suggest an important role of EMT in invasion and metastasis of PDAC and in the development of resistance to standard treatment [14]. Recent evidences suggest that tumor budding could represent one of the morphological hallmarks of EMT [20, 21]. This hypothesis has been recently confirmed by a meta-analysis [22] that investigated the prognostic role of tumor budding and its relationship with the presence of EMT. Authors concluded that high tumor budding is a risk factor for all-cause mortality and they also confirmed that tumor budding is a morphological aspect intimately associated with EMT [22].

Based on transcriptional profile, Collisson *et al*. [23] identified 3 subtypes of PDAC: classical, quasi-mesenchymal and exocrine like. The classical subtype had high expression of adhesion-associated and epithelial genes, the quasi-mesenchymal subtype showed high expression of mesenchyme associated genes and the exocrine-like subtype showed high expression of digestive enzyme genes. Patients with classical subtype showed a better prognosis; each subtype was demonstrated to be an independent prognostic factor for OS. In the pre-clinical setting the quasi-mesenchymal subtype showed better response to gemcitabine [23], however the predictive and prognostic role of this classification has not been tested adequately in the clinical setting and, thus, its application in daily practice is not widely spread.

Waddel *et al*. [24] in 2015 identified 4 subtypes of PDAC depending on chromosomal structural variations: stable, locally rearranged, scattered and unstable, with potential clinical implications. Genomic instability co-segregated with inactivation of DNA maintenance genes (BRCA1; 2) and has possible clinical implications, especially in predicting response to platinum based therapy. Moffitt *et al*. [25] identified two types of PDAC, named “basal-like”, with the worst prognosis, and “classical“ one; interestingly they studied also the stroma, defining 2 types of peri-tumoral stroma: “activated”, which had a poor prognosis, and “normal”. Stromal subtypes were not correlated with tumor subtypes.

## MOLECULAR FACTORS

On the biological side, many efforts were put to identify protein and their level of expression to correlate to OS, PFS and other parameters in order to understand prognostic and predictive factors of this disease.

Besides classical histopathological characteristics, next generation sequencing (NGS) is allowing us to better recognize mPDAC complex mutational landscape [26] and its association to prognosis. KRAS activation occurs in 92% of PDAC; whereas TP53, SMAD4 and CDKN2A inactivation is reported in more than 50% of cases; almost 10% of PDAC harbors mutations in other genes involved in chromatin remodeling and DNA damage repair, like BRCA1 and 2; PALB2 and ATM [27].

The COMPASS trial was proposed in 2015 to identify predictive mutational features in PDAC trough real-time whole genome sequencing (WGS) and RNA sequencing (RNASeq). It is the first translational study to demonstrate that pancreatic cancer has different molecular and genomic subtypes with different response to chemotherapy. The study demonstrated that Moffitt “classical” tumors respond better to first-line chemotherapy compared to those with “basal-like” tumors. GATA6 expression clearly separated “classical” and “basal-like” PDAC subtypes. EGFR overexpression was associated with the basal subtype. The results from COMPASS trial are encouraging, however they should be interpreted with caution: the primary end-point of the study was the feasibility of the genome sequencing in patients with PDAC and not the definition of genomic subtypes; furthermore the sample was small and there were few patients for each molecular subtypes, weakening, thus, the power of the results [28].

In 2017 Connor *et al*. [29] proposed a classification based on genomic instability; they identified 4 groups of patients. The first one displayed deficiencies in the mismatch repair (MMR) system; another group of patients had deficiencies in homologous recombination to repair double strand-breaks. The last two were the “age-related” groups, in which signatures were attributed to mutational processes accumulated over cell divisions. This classification does not carry prognostic value.

The predictive value of these classifications has not been investigated in adequately powered studies.

Several limits apply that hamper the introduction of these analyses in the daily practice: they are costly, they require technological platforms which might not be available, and there is often paucity of available tissue. Furthermore, the molecular subtypes do not correlate with histological variants, contributing to make these classifications difficult to apply on a broad scale.

## MICROSATELLITE INSTABILITY

Microsatellite Instability (MSI) is another molecular feature that can be found in PDAC with an incidence between 1 and 2% [30, 31] and it is typically associated with a germline mutation in MMR (mismatch repair) genes and IHC (immunohistochemistry) loss of MMR expression [32]. The prevalence of dMMR/MSI in PDAC is likely very low, but data in this regard are inconsistent. Hu *et al*. found dMMR in PDAC is a rare event occurring at a frequency of 0.8% (7/833) and all 7 patients with dMMR PDAC were found to have Lynch syndrome [32].

Kim and colleagues conducted a prospective analysis of PD-L1 and MMR IHC on 430 patients (6/430 with PDAC) with advanced gastrointestinal and genitourinary cancers. The dMMR was most common in gastric and colorectal cancer (11.1%), and nearly 0% in PDAC [33]. However, some others have reported MSI/dMMR in PDAC to be as high as 22% [34]. Variabilities in histology, sample sizes, and more so due to non-standardized testing and evolving detection methods could explain this discordance.

The prognostic significance of MSI-H in PDAC is controversial. Some authors did not find prognostic differences between MSI-H and MSS patients [35], while others reported a prolonged survival in MSI-H patients [36]. These contrasting data might be due to the small number of MSI patients. Data about MSI as a predictive factor of response to chemotherapy are based only on a few retrospective studies with a small sample size [37]. Recently, FDA granted the approval of immune checkpoint inhibitors (ICHs) in patients with solid tumors with MSI who failed to respond to first line treatments, including patients with PDAC [38, 39]. This decision was supported by the data of a phase II clinical trial that demonstrated the efficacy of PD-1 blockade in 12 different types of cancer with MSI-H [39].

The widespread use of ICHs in PDAC has met several limitations: MSI-H that is the only approved predictive biomarker in this setting, is rare and it is not routinely tasted; the availability of pathological tissue is often an issue; which form of testing (IHC vs MSI-PCR vs NGS) and measure (MMR-D vs MSI-H vs mutational load) is used to determine the MSI is controversial; many patients with mPDAC are not fit to receive a second line treatment and ICHs use is not refunded in all countries by national regulatory agencies. Further studies are required to find new predictive biomarkers that define better which patients would benefit most from ICHs therapy; to reach this goal adequately powered clinical trial are highly needed. The increasing availability of NGS will help overcoming some of this limitation, giving simultaneously precious prognostic and predictive information.

## GLYPICAN-1 (GPC1)-EXPRESSING CIRCULATING EXOSOMES

Recently, glypican-1 (GPC1)-expressing circulating exosomes were found to be a potential diagnostic tool for PDAC, however their reliability is not well established and their clinical utility is not defined [40]. GPC-1 is a member of heparin sulfate proteoglycans family, its overexpression has been reported to be involved in angiogenesis and tumor growth in pancreatic, cancer, glioma and breast cancer [41]; its prognostic role has been investigated in a study including 178 PDAC patients from the Cancer Genome Atlas (TCGA) and samples from 186 pancreatic cancer patients. Normal pancreatic tissue did not express GPC-1, whereas it was expressed in PDAC, mainly to promote hypomethylation. GPC-1 expression was significantly related with tumor dedifferentiation and diameter. Multivariate analyses indicated that GPC1 was a significant risk factor for poor OS with a 1.82-fold increase in the hazard ratio. Similarly, other authors [41] reported a prognostic role of GPC1 exosomes in 128 PDAC patients at different stages, but they did not describe any diagnostic role of this marker. In conclusion, there are some evidences that GPC-1 exosomes are useful as diagnostic and predictive markers, however their clinical availability is influenced by several limitations: small and non-homogeneous samples, highly specific anti-GPC1 antibodies are not readily available [42].

Circulating ribonucleic acids (RNAs), circulating tumor cell DNA (ctDNA) and circulating tumor cells (CTCs) have been extensively investigate.

## MICRORNA AND LONG NON-CODING RNA

MicroRNAs (miRNAs) and long non-coding RNAs (lncRNAs) represent the two major categories of circulating RNAs with prognostic role in PDAC. MiRNAs are very short chains containing about 22 nucleotides normally involved in the transcriptional and translational machinery [43]. LncRNAs are nucleotidic chains exceeding 200 bases that are not translated into proteins. Besides regulating gene transcription at many levels, they are also specifically involved in the epigenetic regulation of DNA [44].

Evaluation of circulating miRNAs and lncRNAs trough liquid biopsies is appealing and has been extensively performed in a variety of cancers, where these molecules have often shown prognostic and predictive roles [45]. In PDAC, both overexpression and downregulation of specific miRNAs have been associated with poor prognosis. Among others, higher levels of circulating miR-21, miR-155 and miR-221 correlated with outcomes [46–49]. Interestingly, the miRNA 200s family has been shown to suppressor tumor progression via EMT inhibition [50]. More recently, a number of multigene prognostic signatures have been proposed, of 2 [51], 5 [52] or 11 microRNAs [53].

In PDAC, also different types of lncRNAs have been investigated, in both clinical and pre-clinical setting.

Many lncRNAs showed prognostic value, although the reproducibility of the results is controversial. The lncRNAs most consistently associated to shorter OS in PDAC patients are MALAT1 [54–56], AFAP-AS1 [57, 58, 44] and UCA1 [44]. Overall, the individual prognostic value of each lncRNAs is too weak for direct clinical development, therefore more recently panels including analysis of the expression levels of up to 12 lncRNAs are being investigated for their prognostic value [59, 60].

Importantly, reciprocal regulation mechanisms exist between mRNAs, miRNAs and lncRNAs [61]. A bioinformatics approach identified SCAMP1, HCP5, MAL2 and LINC00511 as key lncRNAs regulating competing endogenous RNA sub-network linked to prognosis in PDAC [61]. In a separate study, miR-200c-3p was predicted to be a direct target of MALAT1, and high expression of MALAT1 combined to low expression of miR-200c-3p correlated with shorter OS [62].

Finally, one of the major limitation of most of these studies is that discerning the prognostic from the predictive value has been hampered by the rather homogenous treatment strategies, often involving gemcitabine-based treatments. Among the few circulating RNAs specifically analyzed in patients undergoing FOLFIRINOX treatment, miR-181a-5p has been proposed as a specific prognostic biomarker [63].

## CIRCURLATING TUMOR DNA AND CIRCULATING TUMOR CELLS

Circurlating Tumor DNA (ctDNA) is a subset of circulating extracellular DNA in plasma (also called cell-free DNA, cfDNA), specifically released from cancer cells.

ctDNA may originate from apoptotic and necrotic tumor cells, from living tumor cells, or even from CTCs; thus, it has a variable half-life from 15 minutes up to 2 h [64].

ctDNA, being non-invasively detectable in peripheral blood, has been proposed as potential tumor marker and prognostic factor in many types of cancer. Its clinical applications in PDAC are currently under investigation [65]; these include a role in screening, prognostication via the detection of minimal residual disease, early detection of recurrence, and for patients with advanced disease, tumour genotyping and monitoring treatment response [66].

The prognostic role of ctDNA has been mostly investigated in retrospective studies of patients undergoing surgery; in this subset of subjects, the presence of ctDNA, in particular KRAS mutant ctDNA, in plasma has been associated with worse OS and disease free survival (DFS) [67, 68]. Similarly the detection of ctDNA and the ctDNA variant allelic fraction in patients affected by mPDAC have been reported to be a prognostic factor [69], these findings have, recently, be confirmed by a meta-analysis [70].

The use of ctDNA in clinical practice is promising, but nowadays it cannot be routinely recommended, lacking prospective clinical trials that clearly established the prognostic and predictive value of liquid biopsy in PDAC. Furthermore standardization of sequencing techniques and further development of high-sensitive detection methods are needed [70].

Circulating Tumor Cells (CTCs) are released from primary tumor and/or metastatic sites into the bloodstream, are rare in the circulation and difficult to capture [71], furthermore, the rate of detection varies depending on the sampling site [72]. Studies on the prognostic role of CTCs yielded controversial results [73]; they have been more extensively studied for early detection of PDAC in asymptomatic subjects [74] or as a precocious marker of metastatic disease with different findings [75]. Most of the studies adopted different detection strategies, investigated small and non-homogeneous samples and used different end-points. Given the above issues, our feeling is that the future utility of CTCs is not as promising as that of ctDNA and miRNA.

## CA19-9

The serum carbohydrate antigen CA19-9, a sialylated Lewis blood group antigen, is a tumor-associated antigen that has been shown to be a serum marker for pancreatic cancer [76]. It is the only FDA approved prognostic marker for PDAC [77].

Most of the studies investigating the prognostic role of CA19-9 are retrospective and focus on heterogeneous populations that received preeminently gemcitabine mono-chemotherapy [78–80]. However, up to date, the standard of care for PDAC is a combined regimen with gemcitabine and nab-paclitaxel or FOLFIRINOX. In the ANICE Pac study, the authors retrospectively investigated prognostic factors in patients treated with gemcitabine plus nab-paclitaxel in real life and found normal baseline level of CA19-9 to be an independent prognostic marker for better survival [81].

CA19-9 remains the only biomarker recommended for clinical use by the National Comprehensive Cancer Network (NCCN) guidelines for pancreatic cancer and it is recognized as the most clinically useful biomarker [58]; its sensitivity and specificity are 80% and 80-90% respectively [82], its level is strongly correlated with the tumor burden [83].

Despite its approval, it is far to be an ideal prognostic biomarker: it can yield false negative results in patients who do not express the Lewis blood antigen, accounting from 5% to 10% of general population [82], as it can give false positive results in case of pancreatitis, cholestasis, diabetes, cirrhosis or other cancers [82]. Many studies described the prognostic role of CA19-9 decline after curative surgery [84] and during chemotherapy [85, 86], especially an early decrease during treatment has been supposed to foresee a longer PFS [87] in patients receiving gemcitabine-nab-paclitaxel and FOLFIRINOX. Conversely there are also data that do not support these findings [88].

In patients treated in ACCORD11/PRODIGE4 trial, an early (8 weeks) CA19-9 decrease with respect to 20% was predictive of better PFS and could help to evaluate the efficacy of chemotherapy regimen such as FOLFIRINOX or gemcitabine [87].

Regard OS, a decrease >20% in CA19-9 level during chemotherapy was associated with a longer survival when compared to those with a CA19-9 decrease <20% [86].

It should be underlined that some authors investigated the nadir of CA19-9 response during chemotherapy as a surrogate marker for survival and not as a predictive or prognostic factor [89].

## INFLAMMATORY MARKERS

Chronic inflammation represents both an important etiologic factor in the development of pancreatic cancer as well as a reactive process to pancreatic cancer [90].

The role of systemic inflammation has been studied in several cancers; the most investigated inflammation markers are: C-reactive protein (CRP) and albumin combined as the modified Glasgow Prognostic Score (mGPS), neutrophil- lymphocyte ratio (NLR), platelet-lymphocyte ratio (PLR), white cell count and CRP as the prognostic index (PI) and the combination of albumin and lymphocyte count in prognostic nutritional index (PNI). Those markers have been studied in several solid tumors [91], such as breast cancer [92], hepatocellular carcinoma [93], and colorectal cancer, in both resected [91] and metastatic patients [94].

An increase in inflammatory markers is associated with decreased survival in metastatic pancreatic cancers patients.

The first data about inflammation markers in mPDAC have been reported by Wang *et al*. in 2012 [95]: in 177 patients NLR >5 was associated with worse survival, PLR, mGPS, PI and PNI were not.

Similar results were published by Stotz *et al*. [96] in a cohort of 371 patients: in the unresectable group, the mGPS was associated with poor cancer specific survival only in univariate analysis.

Martin *et al*. [90] retrospectively analyzed the prognostic role of NLR, PLR and mGPS, in 124 PDAC patients (84 with mPDAC and 40 with locally advanced disease) showing that these markers were independent prognostic factors both on univariate as well as multivariate analysis. Liu *et al*. [97] prospectively studied the prognostic role of C-reactive protein (CRP)/albumin (Alb) ratio in 386 PDAC patientsCRP/Alb ratio, diameter of the primary tumor, CA19-9, and TNM stage were incorporated in the multivariate analysis, showing that CRP/Alb ratio, diameter and TNM stage were independent factors for prognosis. When stratified by TNM stage, patients in stages III and IV whose CRP/Alb ratio >0.180 had remarkably poorer OS compared with patients with CRP/Alb ratio <0.180.

Considering patients that received modified FOLFIRINOX, Yamada *et al*. [98], found that the development of neutropenia after chemotherapy correlates with better OS. Moreover, there was a significant correlation between OS and the grade of neutropenia. Similar results have been published for patients receiving gemcitabine monotherapy [99].

There are many reasons that can explain the prognostic role of neutropenia: first of all, as explained above, systemic inflammation plays a tumor promoting role. Furthermore, it has been supposed that myelosuppression occurring in severe neutropenic patients contributes to improve the prognosis of the patients by reducing the level of myeloid-derived suppressing cells, which in advanced stage of cancer, inhibit the anti-tumoral activity of CD4+ T cells.

Yu *et al*. [100] in a cohort of 364 patients affected by advanced or metastatic PDAC receiving gemcitabine-based chemotherapy described an association between high LDH (>185 Iu/mL) and inflammation markers (NLR/LMR/PLR). Poor OS was predicted by tumor stage, CA19-9 levels, serum LDH levels, NLR and lymphocyte/monocyte ratio. Serum LDH levels positively correlated with NLR and PLR, but negatively correlated with LMR.

LDH is a key enzyme in glycolysis, required for the anaerobic conversion of pyruvate to lactate [101]; under physiological conditions, serum LDH concentrations range from 120–250 IU/mL and increase in patients with tumors, liver disease or cardiopathy. LDH levels correlate with tumor burden and may reflect tumor growth and invasive potential [102]. Hypoxia and systemic inflammation are associated with the advanced stages of pancreatic cancer, and serum LDH levels serve as an indirect marker of tumor hypoxia [100]. In a group of patients receiving gemcitabine plus nab-paclitaxel, Hwang *et al*. [103], reported that high mGPS was an independent prognostic factor, whereas, NLR and PLR did not predict OS.

We know that inflammation is pivotal in pancreatic cancer prognosis in many different ways. Common mediators have been shown to regulate both inflammatory and oncogenic pathways involved in the development and disease progression [104]. Both inflammatory and anti-inflammatory cytokines have been studied in pancreatic cancer [104]; cytokines expression correlates negatively with cachessia, sarcopenia and performance status (PS) [105]. Pro-inflammatory cytokines IL-6 and IL-8, as well as anti-inflammatory cytokines IL-10 and tumor growth factor-beta (TGF-β), have been commonly shown to be elevated in pancreatic patients [105]. Mitsunga *et al*. [106] evaluated the prognostic role of Il-6 and IL-1β in 60 patients receiving gemcitabine based chemotherapy for PDAC; they found that high IL-6 and IL1beta before the start of systemic treatment were associated with poorer OS. Similar results have been published by Farren *et al*. [107] on 73 patients with metastatic/inoperable PDAC: they collected serum before the start of chemotherapy and analyzed a panel of 32 cytokines/chemokines. IL-6 and IL-10, were significantly associated with OS. Of the two cytokines, the effect of IL-6 appeared to be dominant. Namely, patients with higher IL-6 levels had worse survival than those with low IL-6, regardless of their IL-10 status. The authors also evaluated the phenotype of circulating immune cells: the expression of CD45RO, a marker of not-naïve T-cells, on CD4+ T cells was significantly associated with OS, though CD45RO expression on CD8+ T cells was not. The frequency of CD8+ T cells expressing the T cell checkpoint molecule CTLA-4 was negatively associated with survival. Paradoxically, CD4+ T cell expression of TIM3, another immunosuppressive molecule, was positively associated with PDAC survival.

Furthermore, systemic inflammation causes metabolic alterations that influence body composition leading to sarcopenia and cachexia, which are recognized to be negative prognostic factors and are known to modify drugs metabolism, increasing the risk of adverse events that require chemotherapy suspension [108]. In clinical practice the most common index used to estimate body composition are BMI and body surface area; other index, as skeletal muscle index (SMI) and skeletal muscle area (SMA) have been demonstrated to provide a better description of body tissues, however they are not widely available, being expensive and time consuming [109]. Furthermore, adjusting chemotherapy dose depending on skeletal muscle did not reduce chemotherapy toxicity in patients treated with gemcitabine plus nab-paclitaxel [110]; nowadays, based on available data, the precise evaluation of body composition is not required to decide the treatment. The prognostic role of body composition has been largely studied in other gastro-intestinal cancers [111]. Some authors have investigated the role of sarcopenia in PDAC prognosis [112], however, due to the limited number of studies available for inclusion, the authors could only conclude that weight loss and sarcopenic obesity might be considered as poor prognostic factors in this disease. A meta-analysis of 11 studies, comprising 2297 PDAC patients in different stages demonstrated that sarcopenia was significantly associated with poorer OS. Sarcopenic obesity was reported in 0.6% to 25.0% of patients and was also significantly associated with poorer OS.

In general, PDAC is an immunologically ‘cold’ tumor due to its low mutational load, dense desmoplasia and rigid extracellular matrix architecture, which restricts the access of effector immune cells to tumor islands; consequently, metastatic pancreatic cancer has shown poor response to immune checkpoint inhibitors [113], since the immune-modulating effect of immune checkpoint inhibitors does not reverse the immune-quiescent environment of pancreatic cancer. Combinations strategies with immune checkpoint inhibitors and other drugs that could alter the immunosuppressive feature of PDAC, like cancer specific vaccines are currently under investigation, but they are far to be available in clinical practice [114].

## NOMOGRAMS AND PROGNOSTIC SCORES

Many nomograms have been validated to predict prognosis in PDAC. One of the strengths of a nomogram is the ability to integrate multiple prognostic factors into a single numerical estimate of survival in an individual patient and thus provides an individualized prediction of survival [115].

Up to date several prognostic models have been proposed in pancreatic cancer, even if in different stages [116–118]. The most widely used prognostic factors included age, sex, tumor size, PS, regional lymph node and distant metastasis, CA19-9 and back pain.

The first nomograms were validated for resected PDAC patients [117] in 2014. Authors prospectively considered a cohort of 531 patients with stage III-IV pancreatic cancer treated with gemcitabine based chemotherapy including six variables: age; sex; PS; tumor size; distant metastasis and regional lymph node.

The nomogram was validated to predict survival probability at 6 - 12- and 18 months, they demonstrated its superiority compared to AJCC TNM staging system in predicting survival.

On the basis of nomogram-predicted survival probabilities, the patients were categorized into the following quartiles of risk: very low (‘Total Points’: 954); low (55–81); high (82–105); and very high (:106). The survival times were significantly differentiated between the groups and the median survival time in the very low-, low-, high-, and very high-risk groups were 17.5 (95% CI: 15.4–22.9), 13.7 (95% CI: 11.6–16.0), 8.9 (95% CI: 7.9–10.4), and 5.5 (95% CI: 4.7–7.5) months, respectively.

In 2016, Vernerey *et al*. [118] proposed a prognostic nomogram for locally PDAC patients, including as parameters: age, pain, tumor size, albumin and CA19-9, which were all independent prognostic factors. Kou *et al*. [119] validated a prognostic model on 306 patients receiving gemcitabine chemotherapy, based on the regression coefficients of the six significant independent negative prognostic factors (PS, distant metastatic disease, the status of recurrent or initially unresectable disease, CEA (≥5.0 ng/ml) and CA19-9 level (≥1,000 U/ml), and neutrophil/lymphocyte ratio (NLR) (≥5).

Recently Hang *et al*. [120] developed and validated a prognostic nomogram considering data from the comparative arm of three trials where metastatic pancreatic patients received gemcitabine monotherapy. They inserted five independent prognostic elements: CA19-9; albumin; absolute neutrophil count (ANC), PS and liver metastasis. Nomogram was validated on an extern cohort to predict survival at 3, 6, 9 and 12 months and patients were divided into 2 groups on the basis of an optimal cut-off value of NTP (nomogram total point) identified by ROC curve: a low risk group (NTP < 109, *N* = 100) and a high risk group (NTP ≥ 109, *N* = 306). The median OS of the low risk group and high risk group was 11.7 (95% CI: 9.7–13.8) and 5.6 months (95% CI: 5.0-6.1), respectively. To give a more specific stratification for patients with high risk, the patients were further categorized into the following tertiles of risk: low risk group (NTP < 111, *N* = 110), intermediate risk group (111 ≤ NTP < 144, *N* = 186) and high risk group (NTP ≥ 144, *N* = 110). The OS was significantly different among the 3 subgroups (P < .001). The median OS was 3.7 months (95% CI: 3.2–4.3) in high risk group, 7.0 (95% CI: 6.4–7.6) months in intermediate risk group and 11.7 (95% CI: 10.1–13.3) months in low risk group, respectively.

In conclusion, nomograms have more accuracy than single factors in predicting prognosis. However, considering the heterogeneity of the samples and the different setting investigated, up to day, there are not sufficient data to recommend a nomogram over another. Furthermore, in clinical practice they are not widely used and international guidelines do not recommend the use of nomograms as predictive tools to guide treatment decisions.

## MECHANISMS OF DRUG RESISTANCE

Molecular pathway involved in drugs pharmacokinetic seems to correlate with OS and PFS. Resistant tumors often have an altered metabolism of gemcitabine. Ohmine *et al*. demonstrated that deoxycytidine kinase (DKC) protein level correlates with PFS in patients treated with gemcitabine. DKC is an enzyme, which converts dFdC (molecular name of gemcitabine) to dFdC monophosphate, contributing to the intracellular activation of the drug. It has demonstrated to be determinant for gemcitabine sensitivity both *in vitro* and *in vivo* [121].

In pancreatic cancer, MUC4 is involved in the acquisition of an aggressive phenotype in the early steps of carcinogenesis [122].

In the study of Shrypek *et al*., the expression of MUC4 in pancreatic cancer cells led to a marked decrease in hCNT1 and hCNT3 transporters which lead gemcitabine inside the tumoral cells, so the expression of this protein could be investigated as potential predictive marker of resistance to chemotherapy regimen containing gemcitabine [123].

The loss of MUC4 was also demonstrated to increase sensibility of cell cultures (CAPAN-2 cells) to 5-fluorouracil. This protein is implicated in the expression level of MRP3 and MRP4, drug-detoxifying channels. While decreased expression of MRP3 did not modify cytotoxicity of gemcitabine, MRP4 repression demonstrated to be linked to a statistically significant increase in survival in pancreatic cancer cell exposed to gemcitabine [122]. Furthermore, MRP expression confers resistance to campotectin, such as irinotecan [124] which is used in the treatment of pancreatic cancer. In PDAC, tumor environment is characterized by hypoxia that contributes to cancer progression and resistance to chemotherapy. Gemcitabine sensitivity in CAPAN-2 (Human Pancreatic Cancer Cell Line) cells was significantly decreased under hypoxia. Overexpression of HIF -1a, a transcription factor expressed to compensate for the hypoxic microenvironment and overexpressed in many tumors, resulted in decreased gemcitabine sensitivity. Chemo-resistance induced by hypoxia is due to the regulation of ABCG2 (ATP-binding cassette subfamily G member 2) which is one of the major multidrug-resistance pumps through the activation of ERK1/2/HIF-1a. ABCG2 could serve as a predictor of gemcitabine response and potentially as a target for chemotherapy of pancreatic cancer.

In patients treated with irinotecan, an association between high expression of CES2, an enzyme that produces irinotecan active metabolite SN-38, and longer survival, was described [125]. ERCC1 overexpression showed to be significantly linked to shorter survival and worse disease control rate, even if in a retrospective study with small sample size [126]. A negative predictive role for ERCC1 was suggested by Mancuso *et al*., who reported that high ERCC1 expression was associated with shorter survival in patients with advanced pancreatic cancer treated with platinum therapy [127].

Data are however not concordant. Tezuka *et al*., even if in small number of patients (34), did not find statistical association between ERCC1, ERCC2, ERCC4, GSTPi and response to FOLFIRINOX [128]. Otherwise, the proteins KRT81 and HNF1A expression showed to be linked to FOLFIRINOX or gemcitabine sensitivity.

The study of Muckenhuber *et al*. [129] suggests that KRT81-positive patients might not derive a relevant advantage from both an intensive chemotherapy regimen as FOLFIRINOX and gemcitabine, while HNF1A-positive patients might benefit from FOLFIRINOX based therapy with a better response compared to gemcitabine treated patients.

hENT1 (Human Equilibrative Nucleoside Trasporter 1) is one of the major gemcitabine transporters and was studied as a predictive factor of response to this drug. Its overexpression is associated with a longer OS and PFS in PDAC [130].

Most of the above reported predictive factors are to be searched on histological samples, whereas novel circulating predictive biomarkers are emerging. Among these, circulating miRNAs are gaining relevance. In particular, focusing on gemcitabine resistance, it was showed, that higher serum mi-RNA 21 and mi-RNA 155 levels were observed in patients that did not obtain response to gemcitabine [131, 48]. However, those data require to be confirmed by broader and adequately powered studies to distinguish better between the prognostic and the predictive role of those markers. In patients receiving FOLFIRINOX for locally advanced or mPDAC, plasma miR-181a-5p was significantly downregulated in non-progressive patients after FOLFIRINOX; this aspect did not correlate with survival in patients treated with gemcitabine plus nab-paclitaxel, confirming the predictive significance of this marker [63].

Given the crescent use of NGS in clinical practice, many previously unknown gene mutations are emerging as predictive markers, potentially targetable by specific innovative therapies. These alterations include recurrent NRG1 rearrangements that drives PDAC development through aberrant ERBB receptor-mediated signaling [132]; the oncogenic DCTN1-ALK fusion and the RRAS mutation that are associated with the development of PDAC in the absence of the KRAS mutation. Constitutional activation of DCTN1-ALK fusion protein was suppressed by the anaplastic lymphoma kinase (ALK) tyrosine kinase inhibitors crizotinib and alectinib. Thus, a small subset of PDAC patients might benefit from therapy using these inhibitors [133]. NTRK fusion is described to be present in <1% of PDAC; this genetic alteration has been specifically targeted by new drugs like entrectinb and larotrectinib that have been tested in basket trials, that select patients independently from the histology, but on the basis of specific molecular alterations. In patients that have received multiple treatments and are fit to be enrolled in clinical trials, it seems reasonable to look for specific, even rare, molecular alterations that make patients eligible to receive targeted therapy. It is worth highlighting, however, that precision medicine is far to be the standard of care for PDAC and nowadays, it is confined to the clinical trial setting.

In Table 3 the main studies which evaluated the molecular pathways involved in drugs resistance or sensibility are summarized.

**Table 3:** Mechanisms of drug resistance

Authors	Study design	Molecules involved	Results
Meijer *et al*. [63]	Microarray-based profiling to discover deregulated miRNAs in pre- and post-chemotherapy plasma according to progression-free survival (PFS) after FOLFIRINOX		Plasma miR-181a-5p was significantly downregulated in non-progressive patients after FOLFIRINOX
Ohmine *et al*. [121]	Analysis of Deoxycytidine kinase (DKC) expression on surgical samples of patients treated with gemcitabine	DKC activates Gemcitabine in PDAC	DKC expression is associated with increased OS in patients receiving Gemcitabine
Shrypek *et al*. [123]	Study of the expression of MUC 4 on PDAC cells	MUC4 downregulates expression of hCNT1 and hCNT3 transporters leading gemcitabine inside tumoral cells	MUC 4 expression in PDAC is associated with gemcitabine resistance
Capello *et al*. [125]	Retrospective analysis of Carboxylesterase 2 (CES2) expression on surgical samples of patients with PDAC that did and did not receive pre-operative chemotherapy	CES2, by mediating the intratumoral activation of irinotecan, is a contributor to FOLFIRINOX sensitivity in pancreatic cancer	Patients treated with FOLFIRINOX with higher expression of CES2 on PDAC samples had better prognosis
Mancuso *et al*. [127]	Retrospective analysis of ERCC1 expression on tissue from patients treated with platinum or fluoropyrimidine based therapy	Excision repair cross complementing 1 (ERCC1) participates to repair mechanism of cisplatin-induced DNA adducts in cancer cells	ERCC1 expression was related to shorter OS in patients receiving platinum-based therapy
Muckenhuber *et al*. [129]	Prospective comparative analysis of Hepatocyte Nuclear Factor-1A and Cytokeratin-81 (HNF1A/KRT81) expression on samples from patients not receiving chemotherapy, receiving FOLFIRINOX or Gemcitabine		Patients with a KRT81-positive subtype did not benefit from FOLFIRINOX therapy, whereas those with HNF1A-positive tumors responded better compared with gemcitabine-based treatment
Song *et al*. [131]	Retrospective analysis of serum expression of mi-RNA 21 in patients affected by PDAC	Histone acetylation levels at miR-21 promoter were increased in PDAC cells after treatment with gemcitabine	Serum miR-21 levels were increased in gemcitabine- resistant PDAC patients compared with gemcitabine-sensitive subjects

The table summarizes the most relevant studies that investigated the mechanisms of drug resistance or sensitivity.

## BRCA 1 AND 2

Pancreatic cancer is the third most common cancer associated with mutations in BRCA genes. BRCA2 and BRCA1 mutations cause respectively a 3.5-10 fold and 2.5 fold higher risk of PDAC. Not by chance, BRCA 2 mutations are described to occur frequently in Familiar Pancreatic Cancer (FPC) [134] and are described in almost 4–7% of patients with PDAC [135].

BRCA genes mutations are associated with defect in homologous recombination repair of DNA double-strand breaks and confer sensibility to poly (adenosine diphosphate-ribose) polymerase (PARP) inhibition [136]. PARP inhibition leads to accumulation of DNA damage and tumor cell death [137].

There are just few data on the use of PARP inhibitors in this disease and they refer to mixed cohorts in early phase trials which showed prolonged partial response for 1-2 years [138, 139]. BRCA mutation is responsible of higher sensitivity to platinum-based therapy. In a retrospective evaluation of 43 patients diagnosed with stage III or IV of BRCA mPDAC, patients treated with platinum-based regimens had median OS that was 13 months longer compared with patients not treated with platinum (median 22 vs 9 months) [140].

Recently Golan *et al*. demonstrated that olaparib maintenance achieved a longer PFS compared to placebo in metastatic pancreatic cancer patients who had a germline BRCA 1 or 2 mutation. Patients eligible for olaparib had received at least 16 weeks of first line platinum-based chemotherapy. mPFS was almost doubled in the experimental group in comparison with placebo group (7.4 months vs 3.8 months) and also difference in median duration of response was significant (24.9 months vs 3.7 months). Even though no benefit in OS was found and the experimental treatment was tested only as maintenance in patients carrying BRCA mutations who had not progressed during first line platinum-based chemotherapy [141]. BRCA germline mutations result a predictive factor of response to PARP inhibitors. Consequently, BRCA testing is likely to spread in everyday clinical practice and represents a potential answer to an unmet clinical need in the field of mPDAC treatment.

## CONCLUSIONS

In a changing landscape consisting in new chemotherapy regimens, immunotherapy and target therapies, the identification of prognostic and predictive factors is needed in view of a personalized medicine which aim to choose the best therapy for the right patient. Further studies are needed to better understand pancreatic cancer biology and to identify prognostic and predictive factors, which could help clinicians to stratify pancreatic cancer patients and improve their prognosis.

## References

[1] Jemal A, Bray F, Center MM, Ferlay J, Ward E, Forman D Global cancer statistics. CA Cancer J Clin. 2011; 61:69–90. 10.3322/caac.20107. 21296855

[2] Siegel R, Naishadham D, Jemal A Cancer statistics, 2013. CA Cancer J Clin. 2013; 63:11–30. 10.3322/caac.21166. 23335087

[3] Varadhachary GR, Tamm EP, Abbruzzese JL, Xiong HQ, Crane CH, Wang H, Lee JE, Pisters PW, Evans DB, Wolff RA Borderline resectable pancreatic cancer: definitions, management, and role of preoperative therapy. Ann Surg Oncol. 2006; 13:1035–46. 10.1245/ASO.2006.08.011. 16865597

[4] Halperin DM, Varadhachary GR Resectable, borderline resectable, and locally advanced pancreatic cancer: what does it matter? Curr Oncol Rep. 2014; 16:366. 10.1007/s11912-013-0366-9. 24445498

[5] Conroy T, Desseigne F, Ychou M, Bouché O, Guimbaud R, Bécouarn Y, Adenis A, Raoul JL, Gourgou-Bourgade S, de la Fouchardière C, Bennouna J, Bachet JB, Khemissa-Akouz F, et al, and Groupe Tumeurs Digestives of Unicancer, and PRODIGE Intergroup FOLFIRINOX versus gemcitabine for metastatic pancreatic cancer. N Engl J Med. 2011; 364:1817–25. 10.1056/NEJMoa1011923. 21561347

[6] Von Hoff DD, Ervin T, Arena FP, Chiorean EG, Infante J, Moore M, Seay T, Tjulandin SA, Ma WW, Saleh MN, Harris M, Reni M, Dowden S, et al Increased survival in pancreatic cancer with nab-paclitaxel plus gemcitabine. N Engl J Med. 2013; 369:1691–703. 10.1056/NEJMoa1304369. 24131140 PMC4631139

[7] Haeberle L, Esposito I Pathology of pancreatic cancer. Transl Gastroenterol Hepatol. 2019; 4:50. 10.21037/tgh.2019.06.02. 31304427 PMC6624347

[8] van Roessel S, Kasumova GG, Verheij J, Najarian RM, Maggino L, de Pastena M, Malleo G, Marchegiani G, Salvia R, Ng SC, de Geus SW, Lof S, Giovinazzo F, et al International Validation of the Eighth Edition of the American Joint Committee on Cancer (AJCC) TNM Staging System in Patients With Resected Pancreatic Cancer. JAMA Surg. 2018; 153:e183617. 10.1001/jamasurg.2018.3617. 30285076 PMC6583013

[9] Allen PJ, Kuk D, Castillo CF, Basturk O, Wolfgang CL, Cameron JL, Lillemoe KD, Ferrone CR, Morales-Oyarvide V, He J, Weiss MJ, Hruban RH, Gönen M, et al Multi-institutional Validation Study of the American Joint Commission on Cancer (8th Edition) Changes for T and N Staging in Patients With Pancreatic Adenocarcinoma. Ann Surg. 2017; 265:185–191. 10.1097/SLA.0000000000001763. 27163957 PMC5611666

[10] Komatsu H, Egawa S, Motoi F, Morikawa T, Sakata N, Naitoh T, Katayose Y, Ishida K, Unno M Clinicopathological features and surgical outcomes of adenosquamous carcinoma of the pancreas: a retrospective analysis of patients with resectable stage tumors. Surg Today. 2015; 45:297–304. 10.1007/s00595-014-0934-0. 24973941

[11] Bilici A Prognostic factors related with survival in patients with pancreatic adenocarcinoma. World J Gastroenterol. 2014; 20:10802–12. 10.3748/wjg.v20.i31.10802. 25152583 PMC4138460

[12] Apte MV, Park S, Phillips PA, Santucci N, Goldstein D, Kumar RK, Ramm GA, Buchler M, Friess H, McCarroll JA, Keogh G, Merrett N, Pirola R, Wilson JS Desmoplastic reaction in pancreatic cancer: role of pancreatic stellate cells. Pancreas. 2004; 29:179–87. 10.1097/00006676-200410000-00002. 15367883

[13] Karamitopoulou E, Zlobec I, Born D, Kondi-Pafiti A, Lykoudis P, Mellou A, Gennatas K, Gloor B, Lugli A Tumour budding is a strong and independent prognostic factor in pancreatic cancer. Eur J Cancer. 2013; 49:1032–39. 10.1016/j.ejca.2012.10.022. 23177090

[14] Kohler I, Bronsert P, Timme S, Werner M, Brabletz T, Hopt UT, Schilling O, Bausch D, Keck T, Wellner UF Detailed analysis of epithelial-mesenchymal transition and tumor budding identifies predictors of long-term survival in pancreatic ductal adenocarcinoma. J Gastroenterol Hepatol. 2015 (Suppl 1); 30:78–84. 10.1111/jgh.12752. 25827809

[15] Nielsen MF, Mortensen MB, Detlefsen S Key players in pancreatic cancer-stroma interaction: cancer-associated fibroblasts, endothelial and inflammatory cells. World J Gastroenterol. 2016; 22:2678–700. 10.3748/wjg.v22.i9.2678. 26973408 PMC4777992

[16] Erkan M, Michalski CW, Rieder S, Reiser-Erkan C, Abiatari I, Kolb A, Giese NA, Esposito I, Friess H, Kleeff J The activated stroma index is a novel and independent prognostic marker in pancreatic ductal adenocarcinoma. Clin Gastroenterol Hepatol. 2008; 6:1155–61. 10.1016/j.cgh.2008.05.006. 18639493

[17] Zhang L, Yao J, Li W, Zhang C Micro-RNA-21 Regulates Cancer-Associated Fibroblast-Mediated Drug Resistance in Pancreatic Cancer. Oncol Res. 2018; 26:827–35. 10.3727/096504017X14934840662335. 28477403 PMC7844724

[18] Prall F Tumour budding in colorectal carcinoma. Histopathology. 2007; 50:151–62. 10.1111/j.1365-2559.2006.02551.x. 17204028

[19] Wartenberg M, Cibin S, Zlobec I, Vassella E, Eppenberger-Castori S, Terracciano L, Eichmann MD, Worni M, Gloor B, Perren A, Karamitopoulou E Integrated Genomic and Immunophenotypic Classification of Pancreatic Cancer Reveals Three Distinct Subtypes with Prognostic/Predictive Significance. Clin Cancer Res. 2018; 24:4444–54. 10.1158/1078-0432.CCR-17-3401. 29661773

[20] Gurzu S, Banias L, Kovacs Z, Jung I Epithelial-mesenchymal transition of tumor budding in colorectal cancer: the mystery of CD44-positive stromal cells. Hum Pathol. 2018; 71:168–69. 10.1016/j.humpath.2017.07.019. 28899739

[21] Wang C, Huang H, Huang Z, Wang A, Chen X, Huang L, Zhou X, Liu X Tumor budding correlates with poor prognosis and epithelial-mesenchymal transition in tongue squamous cell carcinoma. J Oral Pathol Med. 2011; 40:545–51. 10.1111/j.1600-0714.2011.01041.x. 21481005 PMC3135705

[22] Lawlor RT, Veronese N, Nottegar A, Malleo G, Smith L, Demurtas J, Cheng L, Wood LD, Silvestris N, Salvia R, Scarpa A, Luchini C Prognostic Role of High-Grade Tumor Budding in Pancreatic Ductal Adenocarcinoma: A Systematic Review and Meta-Analysis with a Focus on Epithelial to Mesenchymal Transition. Cancers (Basel). 2019; 11. 10.3390/cancers11010113. 30669452 PMC6356259

[23] Collisson EA, Sadanandam A, Olson P, Gibb WJ, Truitt M, Gu S, Cooc J, Weinkle J, Kim GE, Jakkula L, Feiler HS, Ko AH, Olshen AB, et al Subtypes of pancreatic ductal adenocarcinoma and their differing responses to therapy. Nat Med. 2011; 17:500–03. 10.1038/nm.2344. 21460848 PMC3755490

[24] Waddell N, Pajic M, Patch AM, Chang DK, Kassahn KS, Bailey P, Johns AL, Miller D, Nones K, Quek K, Quinn MC, Robertson AJ, Fadlullah MZ, et al, and Australian Pancreatic Cancer Genome Initiative Whole genomes redefine the mutational landscape of pancreatic cancer. Nature. 2015; 518:495–501. 10.1038/nature14169. 25719666 PMC4523082

[25] Moffitt RA, Marayati R, Flate EL, Volmar KE, Loeza SG, Hoadley KA, Rashid NU, Williams LA, Eaton SC, Chung AH, Smyla JK, Anderson JM, Kim HJ, et al Virtual microdissection identifies distinct tumor- and stroma-specific subtypes of pancreatic ductal adenocarcinoma. Nat Genet. 2015; 47:1168–78. 10.1038/ng.3398. 26343385 PMC4912058

[26] Biankin AV, Waddell N, Kassahn KS, Gingras MC, Muthuswamy LB, Johns AL, Miller DK, Wilson PJ, Patch AM, Wu J, Chang DK, Cowley MJ, Gardiner BB, et al, and Australian Pancreatic Cancer Genome Initiative Pancreatic cancer genomes reveal aberrations in axon guidance pathway genes. Nature. 2012; 491:399–405. 10.1038/nature11547. 23103869 PMC3530898

[27] Harada T, Chelala C, Bhakta V, Chaplin T, Caulee K, Baril P, Young BD, Lemoine NR Genome-wide DNA copy number analysis in pancreatic cancer using high-density single nucleotide polymorphism arrays. Oncogene. 2008; 27:1951–60. 10.1038/sj.onc.1210832. 17952125 PMC2492386

[28] Aung KL, Fischer SE, Denroche RE, Jang GH, Dodd A, Creighton S, Southwood B, Liang SB, Chadwick D, Zhang A, O’Kane GM, Albaba H, Moura S, et al Genomics-Driven Precision Medicine for Advanced Pancreatic Cancer: Early Results from the COMPASS Trial. Clin Cancer Res. 2018; 24:1344–54. 10.1158/1078-0432.CCR-17-2994. 29288237 PMC5968824

[29] Connor AA, Denroche RE, Jang GH, Timms L, Kalimuthu SN, Selander I, McPherson T, Wilson GW, Chan-Seng-Yue MA, Borozan I, Ferretti V, Grant RC, Lungu IM, et al Association of Distinct Mutational Signatures With Correlates of Increased Immune Activity in Pancreatic Ductal Adenocarcinoma. JAMA Oncol. 2017; 3:774–83. 10.1001/jamaoncol.2016.3916. 27768182 PMC5824324

[30] Humphris JL, Patch AM, Nones K, Bailey PJ, Johns AL, McKay S, Chang DK, Miller DK, Pajic M, Kassahn KS, Quinn MC, Bruxner TJ, Christ AN, et al, and Australian Pancreatic Cancer Genome Initiative Hypermutation In Pancreatic Cancer. Gastroenterology. 2017; 152:68–74.e2. 10.1053/j.gastro.2016.09.060. 27856273

[31] Lupinacci RM, Goloudina A, Buhard O, Bachet JB, Maréchal R, Demetter P, Cros J, Bardier-Dupas A, Collura A, Cervera P, Scriva A, Dumont S, Hammel P, et al Prevalence of Microsatellite Instability in Intraductal Papillary Mucinous Neoplasms of the Pancreas. Gastroenterology. 2018; 154:1061–65. 10.1053/j.gastro.2017.11.009. 29158190

[32] Hu ZI, Shia J, Stadler ZK, Varghese AM, Capanu M, Salo-Mullen E, Lowery MA, Diaz LA Jr, Mandelker D, Yu KH, Zervoudakis A, Kelsen DP, Iacobuzio-Donahue CA, et al Evaluating Mismatch Repair Deficiency in Pancreatic Adenocarcinoma: Challenges and Recommendations. Clin Cancer Res. 2018; 24:1326–36. 10.1158/1078-0432.CCR-17-3099. 29367431 PMC5856632

[33] Kim ST, Klempner SJ, Park SH, Park JO, Park YS, Lim HY, Kang WK, Kim KM, Lee J Correlating programmed death ligand 1 (PD-L1) expression, mismatch repair deficiency, and outcomes across tumor types: implications for immunotherapy. Oncotarget. 2017; 8:77415–23. 10.18632/oncotarget.20492. 29100397 PMC5652789

[34] Eatrides JM, Coppola D, Al Diffalha S, Kim RD, Springett GM, Mahipal A Microsatellite instability in pancreatic cancer. J Clin Oncol. 2017; 34:e15753–e15753. 10.1200/JCO.2016.34.15_suppl.e15753.

[35] Liu W, Shia J, Gönen M, Lowery MA, O’Reilly EM, Klimstra DS DNA mismatch repair abnormalities in acinar cell carcinoma of the pancreas: frequency and clinical significance. Pancreas. 2014; 43:1264–70. 10.1097/MPA.0000000000000190. 25058881

[36] Yamamoto H, Itoh F, Nakamura H, Fukushima H, Sasaki S, Perucho M, Imai K Genetic and clinical features of human pancreatic ductal adenocarcinomas with widespread microsatellite instability. Cancer Res. 2001; 61:3139–44. 11306499

[37] Riazy M, Kalloger SE, Sheffield BS, Peixoto RD, Li-Chang HH, Scudamore CH, Renouf DJ, Schaeffer DF Mismatch repair status may predict response to adjuvant chemotherapy in resectable pancreatic ductal adenocarcinoma. Mod Pathol. 2015; 28:1383–89. 10.1038/modpathol.2015.89. 26226846

[38] Le DT, Uram JN, Wang H, Bartlett BR, Kemberling H, Eyring AD, Skora AD, Luber BS, Azad NS, Laheru D, Biedrzycki B, Donehower RC, Zaheer A, et al PD-1 Blockade in Tumors with Mismatch-Repair Deficiency. N Engl J Med. 2015; 372:2509–20. 10.1056/NEJMoa1500596. 26028255 PMC4481136

[39] Le DT, Durham JN, Smith KN, Wang H, Bartlett BR, Aulakh LK, Lu S, Kemberling H, Wilt C, Luber BS, Wong F, Azad NS, Rucki AA, et al Mismatch repair deficiency predicts response of solid tumors to PD-1 blockade. Science. 2017; 357:409–13. 10.1126/science.aan6733. 28596308 PMC5576142

[40] Lu H, Niu F, Liu F, Gao J, Sun Y, Zhao X Elevated glypican-1 expression is associated with an unfavorable prognosis in pancreatic ductal adenocarcinoma. Cancer Med. 2017; 6:1181–91. 10.1002/cam4.1064. 28440066 PMC5463070

[41] Zhou CY, Dong YP, Sun X, Sui X, Zhu H, Zhao YQ, Zhang YY, Mason C, Zhu Q, Han SX High levels of serum glypican-1 indicate poor prognosis in pancreatic ductal adenocarcinoma. Cancer Med. 2018; 7:5525–33. 10.1002/cam4.1833. 30358133 PMC6246926

[42] Lai X, Wang M, McElyea SD, Sherman S, House M, Korc M A microRNA signature in circulating exosomes is superior to exosomal glypican-1 levels for diagnosing pancreatic cancer. Cancer Lett. 2017; 393:86–93. 10.1016/j.canlet.2017.02.019. 28232049 PMC5386003

[43] Bartel DP MicroRNAs: target recognition and regulatory functions. Cell. 2009; 136:215–33. 10.1016/j.cell.2009.01.002. 19167326 PMC3794896

[44] Fu XL, Liu DJ, Yan TT, Yang JY, Yang MW, Li J, Huo YM, Liu W, Zhang JF, Hong J, Hua R, Chen HY, Sun YW Analysis of long non-coding RNA expression profiles in pancreatic ductal adenocarcinoma. Sci Rep. 2016; 6:33535. 10.1038/srep33535. 27628540 PMC5024322

[45] López-Urrutia E, Bustamante Montes LP, Ladrón de Guevara Cervantes D, Pérez-Plasencia C, Campos-Parra AD Crosstalk Between Long Non-coding RNAs, Micro-RNAs and mRNAs: Deciphering Molecular Mechanisms of Master Regulators in Cancer. Front Oncol. 2019; 9:669. 10.3389/fonc.2019.00669. 31404273 PMC6670781

[46] Giovannetti E, Funel N, Peters GJ, Del Chiaro M, Erozenci LA, Vasile E, Leon LG, Pollina LE, Groen A, Falcone A, Danesi R, Campani D, Verheul HM, Boggi U MicroRNA-21 in pancreatic cancer: correlation with clinical outcome and pharmacologic aspects underlying its role in the modulation of gemcitabine activity. Cancer Res. 2010; 70:4528–38. 10.1158/0008-5472.CAN-09-4467. 20460539

[47] Karasek P, Gablo N, Hlavsa J, Kiss I, Vychytilova-Faltejskova P, Hermanova M, Kala Z, Slaby O, Prochazka V Pre-operative Plasma miR-21-5p Is a Sensitive Biomarker and Independent Prognostic Factor in Patients with Pancreatic Ductal Adenocarcinoma Undergoing Surgical Resection. Cancer Genomics Proteomics. 2018; 15:321–27. 10.21873/cgp.20090. 29976637 PMC6070706

[48] Mikamori M, Yamada D, Eguchi H, Hasegawa S, Kishimoto T, Tomimaru Y, Asaoka T, Noda T, Wada H, Kawamoto K, Gotoh K, Takeda Y, Tanemura M, et al MicroRNA-155 Controls Exosome Synthesis and Promotes Gemcitabine Resistance in Pancreatic Ductal Adenocarcinoma. Sci Rep. 2017; 7:42339. 10.1038/srep42339. 28198398 PMC5309735

[49] Kawaguchi T, Komatsu S, Ichikawa D, Morimura R, Tsujiura M, Konishi H, Takeshita H, Nagata H, Arita T, Hirajima S, Shiozaki A, Ikoma H, Okamoto K, et al Clinical impact of circulating miR-221 in plasma of patients with pancreatic cancer. Br J Cancer. 2013; 108:361–69. 10.1038/bjc.2012.546. 23329235 PMC3566805

[50] Korpal M, Lee ES, Hu G, Kang Y The miR-200 family inhibits epithelial-mesenchymal transition and cancer cell migration by direct targeting of E-cadherin transcriptional repressors ZEB1 and ZEB2. J Biol Chem. 2008; 283:14910–14. 10.1074/jbc.C800074200. 18411277 PMC3258899

[51] Yu Y, Feng X, Cang S A two-microRNA signature as a diagnostic and prognostic marker of pancreatic adenocarcinoma. Cancer Manag Res. 2018; 10:1507–15. 10.2147/CMAR.S158712. 29942152 PMC6005310

[52] Shi XH, Li X, Zhang H, He RZ, Zhao Y, Zhou M, Pan ST, Zhao CL, Feng YC, Wang M, Guo XJ, Qin RY A Five-microRNA Signature for Survival Prognosis in Pancreatic Adenocarcinoma based on TCGA Data. Sci Rep. 2018; 8:7638. 10.1038/s41598-018-22493-5. 29769534 PMC5955976

[53] Liao X, Han C, Wang X, Huang K, Yu T, Yang C, Huang R, Liu Z, Han Q, Peng T Prognostic value of minichromosome maintenance mRNA expression in early-stage pancreatic ductal adenocarcinoma patients after pancreaticoduodenectomy. Cancer Manag Res. 2018; 10:3255–71. 10.2147/CMAR.S171293. 30233242 PMC6130532

[54] Li L, Chen H, Gao Y, Wang YW, Zhang GQ, Pan SH, Ji L, Kong R, Wang G, Jia YH, Bai XW, Sun B Long Noncoding RNA MALAT1 Promotes Aggressive Pancreatic Cancer Proliferation and Metastasis via the Stimulation of Autophagy. Mol Cancer Ther. 2016; 15:2232–43. 10.1158/1535-7163.MCT-16-0008. 27371730

[55] Pang EJ, Yang R, Fu XB, Liu YF Overexpression of long non-coding RNA MALAT1 is correlated with clinical progression and unfavorable prognosis in pancreatic cancer. Tumour Biol. 2015; 36:2403–07. 10.1007/s13277-014-2850-8. 25481511

[56] Liu JH, Chen G, Dang YW, Li CJ, Luo DZ Expression and prognostic significance of lncRNA MALAT1 in pancreatic cancer tissues. Asian Pac J Cancer Prev. 2014; 15:2971–77. 10.7314/APJCP.2014.15.7.2971. 24815433

[57] Ye Y, Chen J, Zhou Y, Fu Z, Zhou Q, Wang Y, Gao W, Zheng S, Zhao X, Chen T, Chen R High expression of AFAP1-AS1 is associated with poor survival and short-term recurrence in pancreatic ductal adenocarcinoma. J Transl Med. 2015; 13:137. 10.1186/s12967-015-0490-4. 25925763 PMC4458022

[58] Song JY, Chen MQ, Guo JH, Lian SF, Xu BH Combined pretreatment serum CA19-9 and neutrophil-to-lymphocyte ratio as a potential prognostic factor in metastatic pancreatic cancer patients. Medicine (Baltimore). 2018; 97:e9707. 10.1097/MD.0000000000009707. 29369199 PMC5794383

[59] Yu J, Wu WK, Li X, He J, Li XX, Ng SS, Yu C, Gao Z, Yang J, Li M, Wang Q, Liang Q, Pan Y, et al Novel recurrently mutated genes and a prognostic mutation signature in colorectal cancer. Gut. 2015; 64:636–45. 10.1136/gutjnl-2013-306620. 24951259 PMC4392212

[60] Zhang H, Zhu M, Du Y, Zhang H, Zhang Q, Liu Q, Huang Z, Zhang L, Li H, Xu L, Zhou X, Zhu W, Shu Y, Liu P A Panel of 12-lncRNA Signature Predicts Survival of Pancreatic Adenocarcinoma. J Cancer. 2019; 10:1550–59. 10.7150/jca.27823. 31031865 PMC6485218

[61] Wang W, Lou W, Ding B, Yang B, Lu H, Kong Q, Fan W A novel mRNA-miRNA-lncRNA competing endogenous RNA triple sub-network associated with prognosis of pancreatic cancer. Aging (Albany NY). 2019; 11:2610–27. 10.18632/aging.101933. 31061236 PMC6535056

[62] Zhuo M, Yuan C, Han T, Cui J, Jiao F, Wang L A novel feedback loop between high MALAT-1 and low miR-200c-3p promotes cell migration and invasion in pancreatic ductal adenocarcinoma and is predictive of poor prognosis. BMC Cancer. 2018; 18:1032. 10.1186/s12885-018-4954-9. 30352575 PMC6199802

[63] Meijer LL, Garajová I, Caparello C, Le Large TY, Frampton AE, Vasile E, Funel N, Kazemier G, Giovannetti E Plasma miR-181a-5p Downregulation Predicts Response and Improved Survival After FOLFIRINOX in Pancreatic Ductal Adenocarcinoma. Ann Surg. 2018 12 9. 10.1097/SLA.0000000000003084. [Epub ahead of print]. . 30394883

[64] Alix-Panabières C, Pantel K Clinical Applications of Circulating Tumor Cells and Circulating Tumor DNA as Liquid Biopsy. Cancer Discov. 2016; 6:479–91. 10.1158/2159-8290.CD-15-1483. 26969689

[65] Cheng H, Liu C, Jiang J, Luo G, Lu Y, Jin K, Guo M, Zhang Z, Xu J, Liu L, Ni Q, Yu X Analysis of ctDNA to predict prognosis and monitor treatment responses in metastatic pancreatic cancer patients. Int J Cancer. 2017; 140:2344–50. 10.1002/ijc.30650. 28205231

[66] Loft M, Lee B, Tie J, Gibbs P Clinical Applications of Circulating Tumour DNA in Pancreatic Adenocarcinoma. J Pers Med. 2019; 9. 10.3390/jpm9030037. 31323810 PMC6789869

[67] Hadano N, Murakami Y, Uemura K, Hashimoto Y, Kondo N, Nakagawa N, Sueda T, Hiyama E Prognostic value of circulating tumour DNA in patients undergoing curative resection for pancreatic cancer. Br J Cancer. 2016; 115:59–65. 10.1038/bjc.2016.175. 27280632 PMC4931379

[68] Earl J, Garcia-Nieto S, Martinez-Avila JC, Montans J, Sanjuanbenito A, Rodríguez-Garrote M, Lisa E, Mendía E, Lobo E, Malats N, Carrato A, Guillen-Ponce C Circulating tumor cells (Ctc) and kras mutant circulating free Dna (cfdna) detection in peripheral blood as biomarkers in patients diagnosed with exocrine pancreatic cancer. BMC Cancer. 2015; 15:797. 10.1186/s12885-015-1779-7. 26498594 PMC4619983

[69] Strijker M, Soer EC, de Pastena M, Creemers A, Balduzzi A, Beagan JJ, Busch OR, van Delden OM, Halfwerk H, van Hooft JE, van Lienden KP, Marchegiani G, Meijer SL, et al Circulating tumor DNA quantity is related to tumor volume and both predict survival in metastatic pancreatic ductal adenocarcinoma. Int J Cancer. 2020; 146:1445–1456. 10.1002/ijc.32586. 31340061 PMC7004068

[70] Creemers A, Krausz S, Strijker M, van der Wel MJ, Soer EC, Reinten RJ, Besselink MG, Wilmink JW, van de Vijver MJ, van Noesel CJ, Verheij J, Meijer SL, Dijk F, et al Clinical value of ctDNA in upper-GI cancers: A systematic review and meta-analysis. Biochim Biophys Acta Rev Cancer. 2017; 1868:394–403. 10.1016/j.bbcan.2017.08.002. 28801248

[71] Ferreira MM, Ramani VC, Jeffrey SS Circulating tumor cell technologies. Mol Oncol. 2016; 10:374–94. 10.1016/j.molonc.2016.01.007. 26897752 PMC5528969

[72] Bissolati M, Sandri MT, Burtulo G, Zorzino L, Balzano G, Braga M Portal vein-circulating tumor cells predict liver metastases in patients with resectable pancreatic cancer. Tumour Biol. 2015; 36:991–96. 10.1007/s13277-014-2716-0. 25318603

[73] Gasparini-Junior JL, Fanelli MF, Abdallah EA, Chinen LT Evaluating MMP-2 and TGFß-RI expression in circulating tumor cells of pancreatic cancer patients and their correlation with clinical evolution. Arq Bras Cir Dig. 2019; 32:e1433. 10.1590/0102-672020190001e1433. 31038558 PMC6488272

[74] Zhou B, Xu JW, Cheng YG, Gao JY, Hu SY, Wang L, Zhan HX Early detection of pancreatic cancer: Where are we now and where are we going? Int J Cancer. 2017; 141:231–41. 10.1002/ijc.30670. 28240774

[75] Sun Y, Wu G, Cheng KS, Chen A, Neoh KH, Chen S, Tang Z, Lee PF, Dai M, Han RP CTC phenotyping for a preoperative assessment of tumor metastasis and overall survival of pancreatic ductal adenocarcinoma patients. EBioMedicine. 2019; 46:133–49. 10.1016/j.ebiom.2019.07.044. 31375425 PMC6712350

[76] Pleskow DK, Berger HJ, Gyves J, Allen E, McLean A, Podolsky DK Evaluation of a serologic marker, CA19-9, in the diagnosis of pancreatic cancer. Ann Intern Med. 1989; 110:704–09. 10.7326/0003-4819-110-9-704. 2930108

[77] Winter JM, Yeo CJ, Brody JR Diagnostic, prognostic, and predictive biomarkers in pancreatic cancer. J Surg Oncol. 2013; 107:15–22. 10.1002/jso.23192. 22729569

[78] Saad ED, Machado MC, Wajsbrot D, Abramoff R, Hoff PM, Tabacof J, Katz A, Simon SD, Gansl RC Pretreatment CA 19-9 level as a prognostic factor in patients with advanced pancreatic cancer treated with gemcitabine. Int J Gastrointest Cancer. 2002; 32:35–41. 10.1385/IJGC:32:1:35. 12630768

[79] Sezgin C, Karabulut B, Uslu R, Sanli UA, Goksel G, Yuzer Y, Goker E Gemcitabine treatment in patients with inoperable locally advanced/metastatic pancreatic cancer and prognostic factors. Scand J Gastroenterol. 2005; 40:1486–92. 10.1080/00365520510023819. 16293561

[80] Park JK, Yoon YB, Kim YT, Ryu JK, Yoon WJ, Lee SH Survival and prognostic factors of unresectable pancreatic cancer. J Clin Gastroenterol. 2008; 42:86–91. 10.1097/01.mcg.0000225657.30803.9d. 18097296

[81] Fernández A, Salgado M, García A, Buxò E, Vera R, Adeva J, Jiménez-Fonseca P, Quintero G, Llorca C, Cañabate M, López LJ, Muñoz A, Ramírez P, et al Prognostic factors for survival with nab-paclitaxel plus gemcitabine in metastatic pancreatic cancer in real-life practice: the ANICE-PaC study. BMC Cancer. 2018; 18:1185. 10.1186/s12885-018-5101-3. 30497432 PMC6267080

[82] Barhli A, Cros J, Bartholin L, Neuzillet C Prognostic stratification of resected pancreatic ductal adenocarcinoma: Past, present, and future. Dig Liver Dis. 2018; 50:979–90. 10.1016/j.dld.2018.08.009. 30205952

[83] Klapdor R, Bahlo M, Babinski A, Klapdor S CA19-9 serum concentrations—analysis of the serum kinetics during first-line therapy of pancreatic cancer in relation to overall survival. Anticancer Res. 2010; 30:1869–74. 20592394

[84] Glenn J, Steinberg WM, Kurtzman SH, Steinberg SM, Sindelar WF Evaluation of the utility of a radioimmunoassay for serum CA 19-9 levels in patients before and after treatment of carcinoma of the pancreas. J Clin Oncol. 1988; 6:462–68. 10.1200/JCO.1988.6.3.462. 3162513

[85] Halm U, Schumann T, Schiefke I, Witzigmann H, Mössner J, Keim V Decrease of CA 19-9 during chemotherapy with gemcitabine predicts survival time in patients with advanced pancreatic cancer. Br J Cancer. 2000; 82:1013–16. 10.1054/bjoc.1999.1035. 10737382 PMC2374423

[86] Ziske C, Schlie C, Gorschlüter M, Glasmacher A, Mey U, Strehl J, Sauerbruch T, Schmidt-Wolf IG Prognostic value of CA 19-9 levels in patients with inoperable adenocarcinoma of the pancreas treated with gemcitabine. Br J Cancer. 2003; 89:1413–17. 10.1038/sj.bjc.6601263. 14562009 PMC2394360

[87] Robert M, Jarlier M, Gourgou S, Desseigne F, Ychou M, Bouché O, Juzyna B, Conroy T, Bennouna J Retrospective Analysis of CA19-9 Decrease in Patients with Metastatic Pancreatic Carcinoma Treated with FOLFIRINOX or Gemcitabine in a Randomized Phase III Study (ACCORD11/PRODIGE4). Oncology. 2017; 93:367–76. 10.1159/000477850. 28982109

[88] Hess V, Glimelius B, Grawe P, Dietrich D, Bodoky G, Ruhstaller T, Bajetta E, Saletti P, Figer A, Scheithauer W, Herrmann R CA 19-9 tumour-marker response to chemotherapy in patients with advanced pancreatic cancer enrolled in a randomised controlled trial. Lancet Oncol. 2008; 9:132–38. 10.1016/S1470-2045(08)70001-9. 18249033

[89] Ko AH, Hwang J, Venook AP, Abbruzzese JL, Bergsland EK, Tempero MA Serum CA19-9 response as a surrogate for clinical outcome in patients receiving fixed-dose rate gemcitabine for advanced pancreatic cancer. Br J Cancer. 2005; 93:195–99. 10.1038/sj.bjc.6602687. 15999098 PMC2361548

[90] Martin HL, Ohara K, Kiberu A, Van Hagen T, Davidson A, Khattak MA Prognostic value of systemic inflammation-based markers in advanced pancreatic cancer. Intern Med J. 2014; 44:676–82. 10.1111/imj.12453. 24750233

[91] Roxburgh CS, McMillan DC Role of systemic inflammatory response in predicting survival in patients with primary operable cancer. Future Oncol. 2010; 6:149–63. 10.2217/fon.09.136. 20021215

[92] Al Murri AM, Wilson C, Lannigan A, Doughty JC, Angerson WJ, McArdle CS, McMillan DC Evaluation of the relationship between the systemic inflammatory response and cancer-specific survival in patients with primary operable breast cancer. Br J Cancer. 2007; 96:891–95. 10.1038/sj.bjc.6603682. 17375036 PMC2360103

[93] Xiao WK, Chen D, Li SQ, Fu SJ, Peng BG, Liang LJ Prognostic significance of neutrophil-lymphocyte ratio in hepatocellular carcinoma: a meta-analysis. BMC Cancer. 2014; 14:117. 10.1186/1471-2407-14-117. 24559042 PMC4015698

[94] Dell’Aquila E, Cremolini C, Zeppola T, Lonardi S, Bergamo F, Masi G, Stellato M, Marmorino F, Schirripa M, Urbano F, Ronzoni M, Tomasello G, Zaniboni A, et al Prognostic and predictive role of neutrophil/lymphocytes ratio in metastatic colorectal cancer: a retrospective analysis of the TRIBE study by GONO. Ann Oncol. 2018; 29:924–30. 10.1093/annonc/mdy004. 29324972

[95] Wang DS, Luo HY, Qiu MZ, Wang ZQ, Zhang DS, Wang FH, Li YH, Xu RH Comparison of the prognostic values of various inflammation based factors in patients with pancreatic cancer. Med Oncol. 2012; 29:3092–100. 10.1007/s12032-012-0226-8. 22476808

[96] Stotz M, Gerger A, Eisner F, Szkandera J, Loibner H, Ress AL, Kornprat P, AlZoughbi W, Seggewies FS, Lackner C, Stojakovic T, Samonigg H, Hoefler G, Pichler M Increased neutrophil-lymphocyte ratio is a poor prognostic factor in patients with primary operable and inoperable pancreatic cancer. Br J Cancer. 2013; 109:416–21. 10.1038/bjc.2013.332. 23799847 PMC3721392

[97] Liu Z, Jin K, Guo M, Long J, Liu L, Liu C, Xu J, Ni Q, Luo G, Yu X Prognostic Value of the CRP/Alb Ratio, a Novel Inflammation-Based Score in Pancreatic Cancer. Ann Surg Oncol. 2017; 24:561–68. 10.1245/s10434-016-5579-3. 27650825

[98] Yamada Y, Fujii H, Watanabe D, Kato-Hayashi H, Ohata K, Kobayashi R, Ishihara T, Uemura S, Iwashita T, Shimizu M, Suzuki A Severe Neutropenia is Associated with Better Clinical Outcomes in Patients with Advanced Pancreatic Cancer Who Receive Modified FOLFIRINOX Therapy. Cancers (Basel). 2018; 10. 10.3390/cancers10110454. 30453583 PMC6265962

[99] Chen Y, Shi Y, Yan H, Wang YR, Dai GH Timing of chemotherapy-induced neutropenia: the prognostic factor in advanced pancreatic cancer patients treated with gemcitabine/gemcitabine-based chemotherapy. Oncotarget. 2017; 8:66593–600. 10.18632/oncotarget.16980. 29029540 PMC5630440

[100] Yu SL, Xu LT, Qi Q, Geng YW, Chen H, Meng ZQ, Wang P, Chen Z Serum lactate dehydrogenase predicts prognosis and correlates with systemic inflammatory response in patients with advanced pancreatic cancer after gemcitabine-based chemotherapy. Sci Rep. 2017; 7:45194. 10.1038/srep45194. 28345594 PMC5366928

[101] Adams MJ, Buehner M, Chandrasekhar K, Ford GC, Hackert ML, Liljas A, Rossmann MG, Smiley IE, Allison WS, Everse J, Kaplan NO, Taylor SS Structure-function relationships in lactate dehydrogenase. Proc Natl Acad Sci U S A. 1973; 70:1968–72. 10.1073/pnas.70.7.1968. 4146647 PMC433644

[102] Tas F, Aykan F, Alici S, Kaytan E, Aydiner A, Topuz E Prognostic factors in pancreatic carcinoma: serum LDH levels predict survival in metastatic disease. Am J Clin Oncol. 2001; 24:547–50. 10.1097/00000421-200112000-00003. 11801751

[103] Hwang I, Kang J, Ip HN, Jeong JH, Kim KP, Chang HM, Yoo C, Ryoo BY Prognostic factors in patients with metastatic or recurrent pancreatic cancer treated with first-line nab-paclitaxel plus gemcitabine: implication of inflammation-based scores. Invest New Drugs. 2019; 37:584–90. 10.1007/s10637-018-0681-y. 30324344

[104] Roshani R, McCarthy F, Hagemann T Inflammatory cytokines in human pancreatic cancer. Cancer Lett. 2014; 345:157–63. 10.1016/j.canlet.2013.07.014. 23879960

[105] Ebrahimi B, Tucker SL, Li D, Abbruzzese JL, Kurzrock R Cytokines in pancreatic carcinoma: correlation with phenotypic characteristics and prognosis. Cancer. 2004; 101:2727–36. 10.1002/cncr.20672. 15526319

[106] Mitsunaga S, Ikeda M, Shimizu S, Ohno I, Furuse J, Inagaki M, Higashi S, Kato H, Terao K, Ochiai A Serum levels of IL-6 and IL-1β can predict the efficacy of gemcitabine in patients with advanced pancreatic cancer. Br J Cancer. 2013; 108:2063–69. 10.1038/bjc.2013.174. 23591198 PMC3670479

[107] Farren MR, Mace TA, Geyer S, Mikhail S, Wu C, Ciombor K, Tahiri S, Ahn D, Noonan AM, Villalona-Calero M, Bekaii-Saab T, Lesinski GB Systemic Immune Activity Predicts Overall Survival in Treatment-Naïve Patients with Metastatic Pancreatic Cancer. Clin Cancer Res. 2016; 22:2565–74. 10.1158/1078-0432.CCR-15-1732. 26719427 PMC4867263

[108] Schlick K, Magnes T, Ratzinger L, Jaud B, Weiss L, Melchardt T, Greil R, Egle A Novel models for prediction of benefit and toxicity with FOLFIRINOX treatment of pancreatic cancer using clinically available parameters. PLoS One. 2018; 13:e0206688. 10.1371/journal.pone.0206688. 30412592 PMC6226156

[109] Miller J, Wells L, Nwulu U, Currow D, Johnson MJ, Skipworth RJE Validated screening tools for the assessment of cachexia, sarcopenia, and malnutrition: a systematic review. Am J Clin Nutr. 2018; 108:1196–1208. 10.1093/ajcn/nqy244. 30541096

[110] Freckelton J, Croagh D, Holt DQ, Fox A, Wong R, Lee M, Moore GT Body Composition Adjusted Dosing of Gemcitabine-Nab-Paclitaxel in Pancreatic Cancer Does Not Predict Toxicity Compared to Body Surface Area Dosing. Nutr Cancer. 2019; 71:624–28. 10.1080/01635581.2018.1542011. 30741000

[111] Choi MH, Oh SN, Lee IK, Oh ST, Won DD Sarcopenia is negatively associated with long-term outcomes in locally advanced rectal cancer. J Cachexia Sarcopenia Muscle. 2018; 9:53–59. 10.1002/jcsm.12234. 28849630 PMC5803619

[112] Ozola Zalite I, Zykus R, Francisco Gonzalez M, Saygili F, Pukitis A, Gaujoux S, Charnley RM, Lyadov V Influence of cachexia and sarcopenia on survival in pancreatic ductal adenocarcinoma: a systematic review. Pancreatology. 2015; 15:19–24. 10.1016/j.pan.2014.11.006. 25524484

[113] von Bernstorff W, Voss M, Freichel S, Schmid A, Vogel I, Jöhnk C, Henne-Bruns D, Kremer B, Kalthoff H Systemic and local immunosuppression in pancreatic cancer patients. Clin Cancer Res. 2001 (Suppl 3); 7:925s–32s. 11300493

[114] Skelton RA, Javed A, Zheng L, He J Overcoming the resistance of pancreatic cancer to immune checkpoint inhibitors. J Surg Oncol. 2017; 116:55–62. 10.1002/jso.24642. 28628715

[115] Hamada T, Nakai Y, Yasunaga H, Isayama H, Matsui H, Takahara N, Sasaki T, Takagi K, Watanabe T, Yagioka H, Kogure H, Arizumi T, Yamamoto N, et al Prognostic nomogram for nonresectable pancreatic cancer treated with gemcitabine-based chemotherapy. Br J Cancer. 2014; 110:1943–49. 10.1038/bjc.2014.131. 24642625 PMC3992497

[116] Le N, Sund M, Vinci A, Beyer G, Ashan Javed M, Krug S, Neessee A, Schober M, and GEMS collaborating group of Pancreas 2000 Prognostic and predictive markers in pancreatic adenocarcinoma. Dig Liver Dis. 2016; 48:223–30. 10.1016/j.dld.2015.11.001. 26769569

[117] Brennan MF, Kattan MW, Klimstra D, Conlon K Prognostic nomogram for patients undergoing resection for adenocarcinoma of the pancreas. Ann Surg. 2004; 240:293–98. 10.1097/01.sla.0000133125.85489.07. 15273554 PMC1356406

[118] Vernerey D, Huguet F, Vienot A, Goldstein D, Paget-Bailly S, Van Laethem JL, Glimelius B, Artru P, Moore MJ, André T, Mineur L, Chibaudel B, Benetkiewicz M, et al Prognostic nomogram and score to predict overall survival in locally advanced untreated pancreatic cancer (PROLAP). Br J Cancer. 2016; 115:281–89. 10.1038/bjc.2016.212. 27404456 PMC4973163

[119] Kou T, Kanai M, Yamamoto M, Xue P, Mori Y, Kudo Y, Kurita A, Uza N, Kodama Y, Asada M, Kawaguchi M, Masui T, Mizumoto M, et al Prognostic model for survival based on readily available pretreatment factors in patients with advanced pancreatic cancer receiving palliative chemotherapy. Int J Clin Oncol. 2016; 21:118–25. 10.1007/s10147-015-0864-x. 26123314

[120] Hang J, Wu L, Zhu L, Sun Z, Wang G, Pan J, Zheng S, Xu K, Du J, Jiang H Prediction of overall survival for metastatic pancreatic cancer: development and validation of a prognostic nomogram with data from open clinical trial and real-world study. Cancer Med. 2018; 7:2974–84. 10.1002/cam4.1573. 29856121 PMC6051216

[121] Ohmine K, Kawaguchi K, Ohtsuki S, Motoi F, Ohtsuka H, Kamiie J, Abe T, Unno M, Terasaki T Quantitative Targeted Proteomics of Pancreatic Cancer: Deoxycytidine Kinase Protein Level Correlates to Progression-Free Survival of Patients Receiving Gemcitabine Treatment. Mol Pharm. 2015; 12:3282–91. 10.1021/acs.molpharmaceut.5b00282. 26280109

[122] Swartz MJ, Batra SK, Varshney GC, Hollingsworth MA, Yeo CJ, Cameron JL, Wilentz RE, Hruban RH, Argani P MUC4 expression increases progressively in pancreatic intraepithelial neoplasia. Am J Clin Pathol. 2002; 117:791–96. 10.1309/7Y7N-M1WM-R0YK-M2VA. 12090430

[123] Skrypek N, Duchêne B, Hebbar M, Leteurtre E, van Seuningen I, Jonckheere N The MUC4 mucin mediates gemcitabine resistance of human pancreatic cancer cells via the Concentrative Nucleoside Transporter family. Oncogene. 2013; 32:1714–23. 10.1038/onc.2012.179. 22580602 PMC3936121

[124] Tian Q, Zhang J, Tan TM, Chan E, Duan W, Chan SY, Boelsterli UA, Ho PC, Yang H, Bian JS, Huang M, Zhu YZ, Xiong W, et al Human multidrug resistance associated protein 4 confers resistance to camptothecins. Pharm Res. 2005; 22:1837–53. 10.1007/s11095-005-7595-z. 16132345

[125] Capello M, Lee M, Wang H, Babel I, Katz MH, Fleming JB, Maitra A, Wang H, Tian W, Taguchi A, Hanash SM Carboxylesterase 2 as a Determinant of Response to Irinotecan and Neoadjuvant FOLFIRINOX Therapy in Pancreatic Ductal Adenocarcinoma. J Natl Cancer Inst. 2015; 107. 10.1093/jnci/djv132. 26025324 PMC4554193

[126] Strippoli A, Rossi S, Martini M, Basso M, D’Argento E, Schinzari G, Barile R, Cassano A, Barone C ERCC1 expression affects outcome in metastatic pancreatic carcinoma treated with FOLFIRINOX: A single institution analysis. Oncotarget. 2016; 7:35159–68. 10.18632/oncotarget.9063. 27147577 PMC5085217

[127] Mancuso A, Sacchetta S, Saletti PC, Tronconi C, Milesi L, Garassino M, Martelli O, Leone A, Zivi A, Cerbone L, Recine F, Sollami R, Labianca R, et al Clinical and molecular determinants of survival in pancreatic cancer patients treated with second-line chemotherapy: results of an Italian/Swiss multicenter survey. Anticancer Res. 2010; 30:4289–95. 21036754

[128] Tezuka S, Ueno M, Kobayashi S, Morimoto M, Ohkawa S, Hirotani A, Tozuka Y, Moriya S, Nakamura Y, Miyagi Y, Sugimori M, Maeda S Predictive value of ERCC1, ERCC2, ERCC4, and glutathione S-Transferase Pi expression for the efficacy and safety of FOLFIRINOX in patients with unresectable pancreatic cancer. Am J Cancer Res. 2018; 8:2096–105. 30416859 PMC6220150

[129] Muckenhuber A, Berger AK, Schlitter AM, Steiger K, Konukiewitz B, Trumpp A, Eils R, Werner J, Friess H, Esposito I, Klöppel G, Ceyhan GO, Jesinghaus M, et al Pancreatic Ductal Adenocarcinoma Subtyping Using the Biomarkers Hepatocyte Nuclear Factor-1A and Cytokeratin-81 Correlates with Outcome and Treatment Response. Clin Cancer Res. 2018; 24:351–59. 10.1158/1078-0432.CCR-17-2180. 29101303

[130] Giovannetti E, Del Tacca M, Mey V, Funel N, Nannizzi S, Ricci S, Orlandini C, Boggi U, Campani D, Del Chiaro M, Iannopollo M, Bevilacqua G, Mosca F, Danesi R Transcription analysis of human equilibrative nucleoside transporter-1 predicts survival in pancreas cancer patients treated with gemcitabine. Cancer Res. 2006; 66:3928–35. 10.1158/0008-5472.CAN-05-4203. 16585222

[131] Song WF, Wang L, Huang WY, Cai X, Cui JJ, Wang LW MiR-21 upregulation induced by promoter zone histone acetylation is associated with chemoresistance to gemcitabine and enhanced malignancy of pancreatic cancer cells. Asian Pac J Cancer Prev. 2013; 14:7529–36. 10.7314/APJCP.2013.14.12.7529. 24460329

[132] Heining C, Horak P, Uhrig S, Codo PL, Klink B, Hutter B, Fröhlich M, Bonekamp D, Richter D, Steiger K, Penzel R, Endris V, Ehrenberg KR, et al NRG1 Fusions in KRAS Wild-Type Pancreatic Cancer. Cancer Discov. 2018; 8:1087–95. 10.1158/2159-8290.CD-18-0036. 29802158

[133] Shimada Y, Kohno T, Ueno H, Ino Y, Hayashi H, Nakaoku T, Sakamoto Y, Kondo S, Morizane C, Shimada K, Okusaka T, Hiraoka N An Oncogenic ALK Fusion and an RRAS Mutation in KRAS Mutation-Negative Pancreatic Ductal Adenocarcinoma. Oncologist. 2017; 22:158–64. 10.1634/theoncologist.2016-0194. 28167572 PMC5330701

[134] Ghiorzo P Genetic predisposition to pancreatic cancer. World J Gastroenterol. 2014; 20:10778–89. 10.3748/wjg.v20.i31.10778. 25152581 PMC4138458

[135] Holter S, Borgida A, Dodd A, Grant R, Semotiuk K, Hedley D, Dhani N, Narod S, Akbari M, Moore M, Gallinger S Germline BRCA Mutations in a Large Clinic-Based Cohort of Patients With Pancreatic Adenocarcinoma. J Clin Oncol. 2015; 33:3124–29. 10.1200/JCO.2014.59.7401. 25940717

[136] Walsh CS Two decades beyond BRCA1/2: homologous recombination, hereditary cancer risk and a target for ovarian cancer therapy. Gynecol Oncol. 2015; 137:343–50. 10.1016/j.ygyno.2015.02.017. 25725131

[137] O’Connor MJ Targeting the DNA Damage Response in Cancer. Mol Cell. 2015; 60:547–60. 10.1016/j.molcel.2015.10.040. 26590714

[138] Lowery MA, Kelsen DP, Stadler ZK, Yu KH, Janjigian YY, Ludwig E, D’Adamo DR, Salo-Mullen E, Robson ME, Allen PJ, Kurtz RC, O’Reilly EM An emerging entity: pancreatic adenocarcinoma associated with a known BRCA mutation: clinical descriptors, treatment implications, and future directions. Oncologist. 2011; 16:1397–402. 10.1634/theoncologist.2011-0185. 21934105 PMC3228075

[139] Cui Y, Brosnan JA, Blackford AL, Sur S, Hruban RH, Kinzler KW, Vogelstein B, Maitra A, Diaz LA Jr, Iacobuzio-Donahue CA, Eshleman JR Genetically defined subsets of human pancreatic cancer show unique *in vitro* chemosensitivity. Clin Cancer Res. 2012; 18:6519–30. 10.1158/1078-0432.CCR-12-0827. 22753594 PMC3513499

[140] Golan T, Kanji ZS, Epelbaum R, Devaud N, Dagan E, Holter S, Aderka D, Paluch-Shimon S, Kaufman B, Gershoni-Baruch R, Hedley D, Moore MJ, Friedman E, Gallinger S Overall survival and clinical characteristics of pancreatic cancer in BRCA mutation carriers. Br J Cancer. 2014; 111:1132–38. 10.1038/bjc.2014.418. 25072261 PMC4453851

[141] Golan T, Hammel P, Reni M, Van Cutsem E, Macarulla T, Hall MJ, Park JO, Hochhauser D, Arnold D, Oh DY, Reinacher-Schick A, Tortora G, Algül H, et al Maintenance Olaparib for Germline BRCA-Mutated Metastatic Pancreatic Cancer. N Engl J Med. 2019; 381:317–27. 10.1056/NEJMoa1903387. 31157963 PMC6810605

